# Ethical issues of involving people with intellectual disabilities in genomic research: a scoping review protocol

**DOI:** 10.12688/wellcomeopenres.19403.1

**Published:** 2023-08-11

**Authors:** Dorothy Chepkirui, Patricia Kipkemoi, Mary Bitta, Eli Harris, Rosemary Musesengwa, Dorcas Kamuya

**Affiliations:** 1 Health Systems and Research Ethics, KEMRI Wellcome Trust Research Programme, Kilifi, Kenya; 2 Bodleian Health Care Libraries, University of Oxford, Oxford, England, UK; 3 Department of Psychiatry, University of Oxford, Oxford, England, UK; 4 Centre for Tropical Medicine, Nuffield Department of Medicine, University of Oxford, Oxford, England, UK

**Keywords:** Ethical issues, Intellectual disability, neurodevelopmental disorders, genomic research, scoping review

## Abstract

**Background:** Psychiatric genomic research is a growing field of research in Africa that is looking at epigenetics of psychiatric disorders; within which a specific focus is neurodevelopmental disorders including intellectual disability (ID). Conducting this type of research is important to identify etiologies and possible interventions or areas for further research. However, genomic research generally, and psychiatric genomic research, faces many social, ethical, cultural, and legal issues; research involving people with ID is particularly challenging. All research stakeholders - researchers, research review bodies, regulators, patient groups - generally agree that involving people with ID require several considerations, including extra protection. It is also recognized that not involving people with ID in research that is relevant to them means that opportunities to learn on specific issues including lived experiences are missed. In this scoping review, we aim to describe the range of ethical and social-cultural issues concerning involvement of people with intellectual disability in genomic research from existing literature.

**Methods:** This scoping review will be conducted based on the Joanna Briggs Institute guidance for scoping review and reported using the PRISMA-ScR guidelines. Iterative review stages will include systematic search of six databases (Embase, Ovid Global Health, PubMed, Scopus, PsycInfo and Web of Science core collection), screening, charting and synthesis of the data. Forward and backward citation screening will also be done for the articles included in the final review. We will include peer reviewed journal articles, guidance documents and reports. Screening and selection of studies based on the eligibility criteria will be done independently by three reviewers; conflicts will be resolved through discussion with a third reviewer and other experts.

**Results:** The results will be included in the scoping review publication.

**Conclusions:** This scoping review will identify key areas of ethical tensions and possible solutions and inform opportunities for empirical ethics studies.

## Introduction

Intellectual disability (ID) also known as Intellectual Development Disorder, is a disorder with an onset during the development period which affects the intellectual and adaptive functioning. The intellectual domain affects reasoning, learning, judgement, and problem solving, while the adaptive function influence daily life like independent living. The Diagnostic and Statistical Manual of Mental Disorders, Fifth Edition (DSM-5) ^1^ and International Classification of Diseases 11 ^th^ Revision (ICD-11) ^2^ characterise ID based on limitations in the two domains ranging from mild to severe and profound. It is estimated that the prevalence of ID is about 1% globally ^3^, with most of the individuals having the mild form of ID ^2^. According to a meta-analysis by Maulik *et al.,* on population based studies, the prevalence of ID is highest among children and adolescents, and globally this is higher in low and middle income countries (LMICs) compared to high income countries ^4^.

People with ID and other psychiatric disorders are categorised as a vulnerable population in research. Vulnerability, however, consists of complex layers that require in-depth considerations rather than mere labelling ^5^. According to Luna’s framework of vulnerability, it is essential to unpack the different layers of vulnerability while identifying instances where one layer of vulnerability leads to worsening of existing ones or exposure to another, termed as cascade of layers of vulnerability ^5, 6^. People with intellectual disability might have several other layers of vulnerability; it is essential to examine these layers critically to minimize associated risks. Various ethical concerns related to vulnerability of people with ID have been documented including, the unclear decisional capacity to give informed consent which is dependent on severity of ID and complexity of study and the information ^7, 8^. Other documented issues include layers of potential exploitation (where they might be involved in research to access the health care benefits), power disparities between caregivers, people with ID and researchers; issues of guardianship and how best interests can be ascertained ^9^. Specific considerations for people with ID in LMICs include issues around shared decision making (who makes what decision and how this is negotiated within families, how best interests of person with ID are safeguarded and what cultural and social dynamics inform these), and wider implications for involvement in research including consent processes and research participation ^10, 11^. The attraction to health care and compensations offered in research studies can make it difficult to tease out the due and undue inducement in research ^9, 12^. In addition, intellectual disabilities like other neurodevelopmental and psychiatric disorders face many forms of stigma.

ID is commonly caused by genetic factors; genomic and genetic research aim to identify the risk factors and possible aetiology for this disorder ^13, 14^. More specifically in LMICs, there is a dearth of psychiatric genome wide associations studies despite their potential to identify risk factors, aetiology, and possible treatment options for neurodevelopmental disorders ^15^. As highlighted above, there are challenges with involving people with ID in health research. ID has been shown to closely co-occur with other syndromes such as autism spectrum disorder ^16^ and Fragile X syndrome ^17^, which further complicates research involvement. A commentary by de Vries J., in 2019 ^18^ on ethical issues in genomic research in South Africa highlighted the concerns around informed consent, as the return of results is described as a sensitive issue that could further stigmatise the participants. Studies have indicated the value of genomic diagnosis for children with ID ^19^, however according to Lily Hoffman-Andrews ^20^, there is still a huge burden of variants of uncertain significance in clinically care.

This protocol outlines a planned scoping review to understand the range and types of ethical and social cultural issues that arise with involving people with intellectual disability in health research. Due to the dearth of literature from LMICs, we chose a scoping review as a first step to inform planned empirical research in this area. 

### Review question

The scoping review question is; what are the ethical and social-cultural issues concerning involving people with intellectual disability in genomic research? The scoping review will also answer the following specific questions;

1. What ethical issues are arise in involving people with intellectual disability in genomic research and how these issues defined/described?
2. What potential solutions for the ethical issues are provided, and which guidelines, if at all, are drawn on to inform how to respond?
3. What areas of further research or knowledge gaps are identified and what recommendations are outlined in the articles?

## Methods

The proposed scoping review will follow the Joanna Briggs methodology for scoping reviews and the updated revisions of the guidance ^21, 22^. This included a predefined review question, proposed eligibility criteria based on population, concept, context, and key outcome, and search plans as detailed in the sections below.

### Eligibility criteria

Population/participants: this review will focus on research involving; people with intellectual disabilities, adults and children; family and caregivers of people with ID and research on perspectives of researchers of studies involving people with ID. ID will be used in this study alongside other syndromes and disorders that lead to cognitive impairment. This study will exclude any study on ethical issues in genetic or prenatal screening and counselling since these are usually conducted within a clinical setting and thus beyond the scope of our work which focuses on research. We will also exclude articles on genomic research reporting ethical issues for general population and not specific to ID.

Context: Articles in English language will be included due to resource constrains within the team to do translations. There will be no limititation in year of research or publication for the articles to be screened and included. We will do a second layer of screening where we will segregate the included articles based on the country where the study was conducted. The aim will be to focus on findings from LMICs as this will ensure the issues raised are relevant to our context in Kenya.

Key outcomes: Themes regarding ethical issues and opinions on ethical concerns when involving people with intellectual disabilities in genetic and/ genomic research. Proposed solutions to these ethical issues will be identified and reported, additionally key areas for future research and knowledge gaps reported in the included studies will be extracted.

### Searches

This review will follow a comprehensive search strategy to identify literature from online databases and extensive hand searching. Key words such as ‘ethics’, ‘principle’, ‘intellectual disability’, ‘genomic research’, and their synonyms will be entered to the databases. An initial preliminary search will be conducted in PubMed focusing on the medical subject headings, then the key articles from this search used to expand the search strategy. The second stage will involve systematic search of the other databases based on adaptation of the search strategy since the indexing terms might be different. The target databases will be Ovid Embase, Ovid Global Health, PubMed, Scopus, Ovid PsycInfo and Web of Science core collection. The sample Ovid Embase search strategy is presented in Box 1 below. Articles from these searches will be exported for title and abstract screening. The last stage will involve forward and backward citation screening of the articles that will be selected for inclusion in the systematic review.

**Box 1. Ovid Embase search strategy**

**#, Query, Results from 8 Feb 2023**

1 exp ethics/ 340,930

2 (ethic* or moral* or principle* or bioethic* or bio-ethic*).ti,ab,kw., 604,546

3 1 or 2 811,263

4 exp genomics/ 136,473

5 ("human genome" or hapmap or genomic* or (genetic* adj6 research*)).ti,ab,kw. 487,244

6 4 or 5 1,604,598

7 exp intellectual impairment/ 590,396

8 ("intellectual disabilit*" or "intellectual dysfunction*" or "intellectual development disorder*" or "mental retard*" or "mental deficien*" or "cri-du-chat syndrome" or "de lange syndrome" or "down syndrome" or "Prader-Willi Syndrome" or "Rubinstein-Taybi Syndrome" or "Trisomy 13 Syndrome" or "WAGR Syndrome" or "Williams Syndrome" or "fragile x").ti,ab,kw.

### Screening and data extraction

Articles will be exported to EndNote reference manager and deduplication using Systematic review accelerator deduplicator programme SR-Accelerator ( https://sr-accelerator.com/#/deduplicator). These will then be exported to Rayyan for title and abstract screening. Screening will be done by the primary reviewer (DC) to assess whether they meet the inclusion criteria outlined above. A second independent reviewer (DK) will screen the included articles to verify inter-rater reliability of the process. An effort will be made to obtain full texts for articles that are unclear if they meet the inclusion criteria, expert consultation will also be done at this instance. Those included at this stage will be screened by full text against the inclusion criteria by the reviewer (DC), and 20% of these screened independently by the second reviewer (DK). Any disagreements will be resolved through expert consultation and by the third independent reviewer (RM, PK, and MB). A list of reasons for exclusion will be compiled at this stage and reported as part of the final systematic review. The results of the screening and evidence selection will be presented using the Preferred Reporting Items for Systematic Reviews and Meta-analyses extension for scoping review (PRISMA-ScR) ^23^

Data extraction of the included articles will be done based on a piloted tool developed *a priori* ( Table 1). Data extraction information will include, study design and setting, country, participant characteristics/population, data collection methods, ethical issues and how they were addressed, future recommendations for conducting such research and research gaps. The draft tool will be amended, if need be, during the extraction process.

**Table 1.** Data extraction template.

VARIABLE	DESCRIPTION
**Author(s), year**	Author(s) and year of publication
**Title**	Title
**Type of article**	Indicate type for example, journal article, guidance document, commentary, conference notes, report, policy etc
**Study design**	Indicate design used to collect data on the ethical issues e.g., interviews, anthropological exploration, meetings, discussions, commentary etc
**Country **	Country where the data was collected or where mentioned, where the findings apply
**Population(s)**	Indicate the population demographics mentioned in the article
	1. Age
	2. Gender
**Neuropsychiatric disorder(s)**	Indicate disorders including ID, relating to the ethical issues raised e.g., Downs Syndrome, Fragile X etc
**Ethical issues described**	Record the ethical issues as described in the article either from findings, discussions, or conclusion sections
**Proposed solutions**	Indicate proposed solutions to the ethical issues raised
**Knowledge gaps in addressing the ** **ethical concerns**	Based on conclusions and recommendation, indicate the gaps
**Comments **	Note any relevant comments relating to the article/findings/key considerations

### Data synthesis and analysis

A summary of the extracted data will be presented in a table adapted from the data extraction tool. An accompanying narrative synthesis will be presented for the articles included to map out the ethical issues in involving people with intellectual disability, recommendations for further research and knowledge gaps. We will synthesise the emerging ethical issues and present the key themes in a narrative format. The findings will be presented in the final publication of the scoping review and shared through presentations in different fora.

## Conclusion

This review is pivotal for research conduct in low- and middle-income countries. This is specifically important for genomic studies known for its complexity in terminology and added layers of intellectual disabilities. We aim to identify key areas of ethical tensions and possible solutions and inform opportunities for empirical ethics studies within our contexts.

## Data Availability

No data are associated with this article.
