# Supplementary material for: Ethical issues of involving people with intellectual disabilities in genomic research: a scoping review protocol
Source: Wellcome Open Res. 2023 Aug 11;8:340. [Version 1] doi: 10.12688/wellcomeopenres.19403.1 (PMC10624948; doi:10.12688/wellcomeopenres.19403.1)
Supplement: Peer review history

# Reviewer report: Reviewer response for version 1

**DOI:** 10.21956/wellcomeopenres.21492.r68760

**Published:** 2023-11-03

**Authors:** Megan Campbell

**Affiliations:** 1 Psychology Department, Rhodes University, Grahamstown, Eastern Cape, South Africa

Thank you for the opportunity to review this important work.

 The article aims to conduct a scoping review of the key ethical and social/cultural issues impacting people with ID participating in genomics research. 

 The introduction provides an appropriate overview of ID; then considers the issue of vulnerability within research and how this would pertain to people with ID. It concludes with an overview of ethical concerns in genomics research and how these may impact on people with ID participating in such research.

- Considering that the review seeks to understand ethical and social/cultural issues, particularly in LMIC settings that are similar to Kenya, a short paragraph on some of these anticipated social/cultural factors and how they may impact on people living with ID and their participation in genomics research would be useful.  

The article then outlines the key aim and specific scoping review questions which are well articulated.

- It would be useful to decide on whether you are addressing ethical, social and cultural issues OR ethical, social-cultural issues OR ethical, and socio-cultural issues, with a short explanation of how you are defining these. You use different terms in different paragraphs  

The methods section is particularly well done with a thorough description of the planned scoping review process.

 There are small grammatical errors throughout which should be corrected.

Is the study design appropriate for the research question?

Yes

Is the rationale for, and objectives of, the study clearly described?

Yes

Are sufficient details of the methods provided to allow replication by others?

Yes

Are the datasets clearly presented in a useable and accessible format?

Not applicable

Reviewer Expertise:

Ethics of neuropsychiatric genomics research.

I confirm that I have read this submission and believe that I have an appropriate level of expertise to confirm that it is of an acceptable scientific standard.

# Reviewer report: Reviewer response for version 1

**DOI:** 10.21956/wellcomeopenres.21492.r68764

**Published:** 2023-11-01

**Authors:** Signe Mežinska

**Affiliations:** 1 University of Latvia, Riga, Latvia

Thank you for the opportunity to review this scoping review protocol. I agree with the authors that the ethical aspects of involving people with intellectual disabilities in genomic research in LMIC are very important. A scoping review is an appropriate methodology for the aims envisaged by the authors. To improve the protocol, I would suggest considering the following issues:

1. The focus of the review is not completely clear. In the introduction and methodology parts the authors several times emphasize that the focus will be on LMIC. At the same time, this focus is not included in the title and research questions. It would be advisable to include the LMIC focus in the title and research questions or to explain how the focus on LMIC will be implemented. A clear focus is encouraged also by the Joanna Briggs methodology for scoping reviews especially by ‘‘PCC’’ mnemonic (population, concept, and context). It seems to me, that LMIC is the “context” in this case.
2. Please, reconsider the review question no. 1 “ *What ethical issues are arise in involving people with intellectual disability in genomic research and how these issues defined/described?*”. The question is not clear and includes grammatical inconsistencies.
3. Please, reconsider the review question no. 3 *“What areas of further research or knowledge gaps are identified and what recommendations are outlined in the articles?” *Do you mean recommendations regarding further research here? If not, then my suggestion would be to delete the second part of the sentence and formulate the question in the following form “ *What areas of further research or knowledge gaps are identified?*”. The topic of recommendations (if you do not mean recommendation for further research) might be included in question no. 2 which already addresses the topic of potential solutions.
4. It is not clear, whether the authors plan to include in the scoping review only primary empirical research studies published in peer-reviewed journals as it seems based on “Eligibility criteria”, or also other types of articles, as mentioned in the abstract (mentioning “guidance documents” and “reports” not mentioned in the further text) and  the Data Extraction Template which mentions also “meetings”, “discussions”, “commentaries”. Additionally, do the authors plan to include reviews or meta-analyses?  Will only articles published in peer-reviewed journals or also book chapters, conference abstracts and publications, grey literature etc. be included? Please clarify the type of articles you plan to include in your scoping review by providing more clear inclusion and exclusion criteria.
5. The authors mention that they will apply “extensive hand searching” additionally to the data base search. The Joanna Briggs methodology requires that hand search must be detailed, e.g., by including journal names and years searched. Please, detail the hand search you are planning.
6. In the Data Extraction Template, line 4 “Study design”, it would be useful to introduce more consistent categories of study design, e.g., qualitative methods (interviews, focus group discussions, observations, ethnographies etc.), quantitative methods (surveys etc.), mixed methods etc. I would doubt whether “commentary” might be included as a type of study design (it might be a type of article, as already mentioned in the previous line). Also, “meeting” and “discussions” is not a study type.
7. Please explain what the sentence “A second independent reviewer (DK) will screen the included articles to verify inter-rater reliability of the process” in the “Screening and data extraction” part means. Do you mean title and abstract screening by the second reviewer?  Why will the second reviewer screen only the included articles? How will this process allow for verifying inter-rater reliability? What if there might be different opinions regarding excluded articles?

Is the study design appropriate for the research question?

Yes

Is the rationale for, and objectives of, the study clearly described?

Yes

Are sufficient details of the methods provided to allow replication by others?

No

Are the datasets clearly presented in a useable and accessible format?

Not applicable

Reviewer Expertise:

Research ethics, bioethics, empirical bioethics

I confirm that I have read this submission and believe that I have an appropriate level of expertise to confirm that it is of an acceptable scientific standard, however I have significant reservations, as outlined above.

# Reviewer report: Reviewer response for version 1

**DOI:** 10.21956/wellcomeopenres.21492.r68761

**Published:** 2023-10-27

**Authors:** Johan Thygesen, Jung Won Choi

**Affiliations:** 1 Institute of Health Informatics, University College London, London, England, UK

This proposed scoping review protocol focuses on the important and complicated topic of the ethics of conducting genomics research in potentially vulnerable people with intellectual disabilities. The researchers a proposing a specific focus on the impact of this type of research within low- and middle-income countries. I believe this is a really important research subject and that the suggested methods for the scoping review are clear and appropriate and should give valuable insights to the community. I only have minor suggestions for clarifications as suggested below.

 Minor:

 Under review question (The wording of the specific questions could be improved).

1.  move “are”, further down?.
2. Consider replacing “if at all” -> “if any”
3. Which main areas of further research/knowledge gaps and recommendations are outlined in the articles?
Under context: The second layer of segregating studies based on the country with a focus on LMICs, is meaningful, but it was unclear to me if this was an inclusion criteria for all studies in general, or if you will analyse all studies (no matter country of origin) and then specifically consider your specific research questions, with focus on both LMIC studies and none-LMIC studies. It would be good to clarify this point.

Is the study design appropriate for the research question?

Yes

Is the rationale for, and objectives of, the study clearly described?

Yes

Are sufficient details of the methods provided to allow replication by others?

Yes

Are the datasets clearly presented in a useable and accessible format?

Yes

Reviewer Expertise:

Genetics, Rare-variants, EHR

We confirm that we have read this submission and believe that we have an appropriate level of expertise to confirm that it is of an acceptable scientific standard.
